# MicroRNA‐433‐3p enhances chemosensitivity of glioma to cisplatin by downregulating NR5A2

**DOI:** 10.1002/brb3.2632

**Published:** 2022-10-27

**Authors:** Jun Li, Jingshun Gu, Juntong Wang, Aiwu You, Yuyan Zhang, Guomin Rao, Shuang Li, Xuehua Ge, Kun Zhang, Dongchun Wang

**Affiliations:** ^1^ The Fourth Department of Neurosurgery Tangshan Gongren Hospital Tangshan China; ^2^ The Fourth Department of Neurology Tangshan Gongren Hospital Tangshan China; ^3^ Department of Traditional Chinese Medicine Tangshan Gongren Hospital Tangshan China

**Keywords:** glioma, malignant progression, microRNA‐433‐3p, NR5A2

## Abstract

**Objective:**

We attempted to investigate influence of microRNA‐433‐3p on malignant progression of glioma and identify its molecular mechanism, thus laying groundwork for glioma management.

**Methods:**

Expression data along with clinical data of glioma were accessed from the TCGA database for differential and survival analyses to look for the target differentially expressed genes. Quantitative reverse transcriptase PCR (qRT‐PCR) and western blot were utilized to assess NR5A2 mRNA and protein expression in different glioma cell lines, respectively. MTT, Transwell assay, and flow cytometry were carried out to assay the impact of NR5A2 on behaviors of glioma cells in vitro. Bioinformatics analysis was used to identify the upstream microRNA of NR5A2 in glioma, while dual‐luciferase and western blot assays were used to detect binding of microRNA and NR5A2. Chemosensitivity of glioma cells was evaluated by cisplatin cytotoxicity test.

**Results:**

NR5A2 was upregulated in both glioma tissues and cell lines. Dual‐luciferase assay result showed binding site of microRNA‐433‐3p on NR5A2 mRNA 3′UTR, and microRNA‐433‐3p reduced NR5A2 expression. Cell assays revealed that silencing NR5A2 could hamper proliferation, invasion, and migration and enhance chemosensitivity to cisplatin while promoting glioma cell apoptosis and blocking glioma cells in G0/G1 phase. Rescue experiments also indicated that microRNA‐433‐3p suppressed glioma malignant progression via inhibiting NR5A2.

**Conclusion:**

MicroRNA‐433‐3p which is significantly poorly expressed in glioma targets NR5A2 to suppress glioma malignant progression and enhance chemosensitivity to cisplatin.

## Graphical abstract

miR‐433‐3p mediated the ability of NR5A2 to regulate the malignant behavior of glioma cells

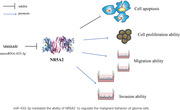

## 1 INTRODUCTION

Glioma is a prevalent malignant tumor (Zhang et al., 2019 ), mainly including two subtypes: diffuse and nondiffuse glioma (Khani et al., 2019). It grows rapidly, leading to low survival rate and high mortality, and it is a serious threat to human life (Chen et al., 2019; Van Meir et al., 2010). Glioma always occurs in the brain. Glioma accounts for 30% of brain and central nervous system tumors and 80% of all brain malignancies (de Robles et al., 2015; Ostrom et al., 2014). Despite great efforts in combination therapy of glioma over the past decades, the prognosis is still unfavorable (Fukushima et al., 2013). In the treatment of malignant glioma, cisplatin has been utilized as an adjuvant therapy of carmustine and a neoadjuvant therapy of temozolomide (Y. Wang et al., 2017). But in clinical application, gliomas are less sensitive to cisplatin, and high doses of cisplatin lead to adverse reactions, which is a shortcoming of platinum‐based drugs in the management of gliomas (L. Yang et al., 2018). Hence, understanding molecular markers related to chemoresistance, so as to enhance the anticancer efficacy of cisplatin‐based treatments, is key to clinical cancer treatment. This study aimed to explore the pathogenesis and drug resistance mechanism of glioma and lay groundwork for clinical diagnosis and management of glioma in the future.

NR5A2 is a structurally alive orphan nuclear receptor (Stergiopoulos & Politis, 2016) that plays an important role in early development via modulating the key pluripotent factor Oct4 level in embryonic stem cells (Gu et al., 2005; Heng et al., 2010). NR5A2 is also an important mediator for liver metabolic balance (Goodwin et al., 2000; Lu et al., 2000; Sirianni et al., 2002). In addition, it is also involved in glucose metabolism, acini differentiation, and other processes (Cobo et al., 2018). In the field of tumor research, it has been reported that NR5A2 as an oncogene is involved in the development of breast cancer (S. Wang et al., 2018), gastric cancer (Liu et al., 2019), prostate cancer (Xiao et al., 2018), and pancreatic cancer (Hale et al., 2014). NR5A2 is implicated in cancer cell chemosensitivity. For example, microRNA‐27b‐3p directly targets NR5A2 and CREB1 to enhance tamoxifen‐induced breast cancer cell toxicity (Zhu et al., 2016). Dual‐inhibition of NR5A2‐GATA6 and BCL‐XL increases sensitivity of esophageal adenocarcinoma cells to cisplatin therapy (Duggan et al., 2016). Although NR5A2 plays a pivotal role in affecting tumor cell chemosensitivity, the role of NR5A2 in glioma and its molecular mechanism are still unclear.

We investigated the mechanism of NR5A2 underlying malignant progression of glioma through assays and further studied the sensitivity of glioma to cisplatin, laying groundwork for future clinical diagnosis and management.

## 2 MATERIALS AND METHODS

### 2.1 Bioinformatics methods

Data of mature microRNAs (normal: 5; tumor: 530) and mRNAs (normal:5; tumor:698) at expression level were downloaded from the TCGA database. Expression differences in normal and glioma tissues were assessed by *t*‐test, with benign tissue as control. R package “survival” was utilized to conduct survival analysis on NR5A2. R package “edgeR” (|logFC| > 2, padj < 0.01) was conducted to screen differentially expressed microRNAs (DEmicroRNAs). R package “survival” was utilized to conduct survival analysis on DEmicroRNAs. To select the microRNA of interest that had significant correlation with NR5A2, miRDB (http://mirdb.org/), starBase (http://starbase.sysu.edu.cn/), and mirDIP (http://ophid.utoronto.ca/mirDIP/index.jsp) databases were used to predict microRNAs regulated by NR5A2 in upstream, which were overlapped with downregulated DEmicroRNAs.

### 2.2 Cell culture and transfection

Human normal glial cell line HEB (BNCC338123) and human glioma cell lines T98G (BNCC338721) were accessed from BeNa Culture Collection. HS683, U87, and glioma resistant cell lines HS683/DDP and U87/DDP were purchased from KeygeneBio (Nanjing, China). Cell lines were grown in DMEM with 10% fetal bovine serum (FBS) at 37°C and 5% CO_2_.

MicroRNA‐433‐3p mimic (100 nmol/L), microRNA‐433‐3p inhibitor (100 nmol/L), vectors with overexpressed NR5A2 (oe‐NR5A2) (100 nmol/L), small interfering RNAs (siRNAs) targeting NR5A2 (si‐NR5A2) (100 nmol/L), and negative control (NC mimic, NC inhibitor, oe‐NC, si‐NC) were synthesized by Gima (Shanghai, China). Cells were transfected with the Lipofectamine 2000 transfection reagent (Invitrogen, USA).

### 2.3 qRT‐PCR

Total RNA from glioma cells was separated by Trizol (Invitrogen). Complementary DNA was reversely transcribed with the PrimeScript RT kit, and qRT‐PCR) was done with the SYBR Premix Ex TaqTM II kit on the Roche LC480 real‐time quantitative PCR system (Roche). The conditions for qRT‐PCR were as follows: 95°C for 10 s, 60°C for 20 s, 72°C for 30 s, 45 cycles, and qRT‐PCR was conducted using a Roche Lightcycler 480 real‐time PCR machine. U6 and GAPDH were applied as endogenous controls for microRNA and mRNA expression analysis, respectively. The 2^‐ΔΔCt^ method was used for normalizing the results. Primer sequences shown in Table 1 were synthesized by RiboBio company (Guangzhou, China). The assay was repeated three times.

**TABLE 1:** Primer sequences

Gene	Sense sequence (5′−3′)	Antisense sequence (5′−3′)
microRNA‐433‐3p	GGAGAAGTACGGTGAGCCTGT	GAACACCGAGGAGCCCATCAT
NR5A2	GGGTACATTATGGGCTCCT	TGTCAATTTGGCAGTTCTGG
U6	CTCGCTTCGGCAGCACA	AACGCTTTCACGAATTTGCGT
GAPDH	GTGTTCCTACCCCCAATGTG	CATCGAAGGTGGAAGAGTGG

### 2.4 MTT assay

Cell viability was examined by MTT assay. Glioma cells were plated in 96‐well plates with 5 × 10^3^ cells/well 12 h before transfection. MTT assay was introduced after transfection for 24, 48, and 72 h. Specifically, 10 μl MTT (5 mg/ml) was added to each well at the above time points for cell incubation in a 37°C incubator for 4 h. After supernatant was discarded, 200 μl dimethyl sulfoxide was added. The optical density (OD) values at 490 nm were measured using a microplate reader (ThermoFisher Scientific, USA) to examine cell viability. The assay was repeated three times. Cell growth curves were drawn with time as the horizontal axis and the ratio of absorbance values of cells in each group at each time point as the vertical axis.

### 2.5 Flow cytometry assay

Glioma cells were plated into 6‐well plates, and the Annexin V‐FITC apoptosis assay kit (BD Biosciences, USA) was implemented to evaluate apoptosis of cells in different treatment groups. At room temperature, cells were digested by trypsin and washed. Afterward, the FACSCanto Ⅱ flow cytometry instrument (BD Biosciences) was applied for cell apoptosis analysis, with the FITC‐labeled Annexin V as the fluorescence probe. The assay was repeated three times.

FACSCanto Ⅱ flow cytometry instrument (BD Biosciences, USA) was introduced for cell cycle assay at 48 h after mimic transfection. Note that 1 × 10^6^ cells were collected, followed by washing with cold PBS, and fixing with 70% cold ethanol at 4°C for 48 h. Next, after rinsing cells with cold PBS again, propidium iodide (PI) was used for staining nucleus followed by measuring DNA amount using flow cytometry. At least 1 × 10^4^ cells were counted, and the cell cycle distribution was analyzed using ModFit software.

### 2.6 Transwell migration and invasion assays

In cell migration assay, 5 × 10^5^ glioma cells were added into the top insert without coating Matrigel. In the cell invasion assay, 1 × 10^5^ glioma cells were added into the top chamber coated with Matrigel matrix. Cells were added into the top insert with serum‐free medium, and medium with 20% serum (Gibco, USA) was added into the bottom chamber as a chemical inducer in both assays. The cells were maintained under routine conditions. After 16 h, the cells on the surface of the membrane were removed with a cotton swab, the migrated/invading cells under the membrane were stained with crystal violet, and five areas were randomly selected for photographing and cell counting.

### 2.7 Western blot assay

After transfection for 48 h, cells were lysed in radioimmunoprecipitation buffer (Solaibo, China) to extract total proteins. BCA protein assay kit was utilized to quantify the proteins. The proteins were dispersed by sodium dodecyl sulfate‐polyacrylamide gel electrophoresis (SDS‐PAGE), followed by transferring the proteins to a polyvinylidene fluoride (PVDF) membrane. The membrane was sealed with 5% skim milk at room temperature for 1 h and maintained with relevant antibodies overnight. After incubation with secondary antibodies (horseradish peroxidase‐conjugated IgG), protein bands were developed by ECL. Finally, Image J software was used to quantify the protein bands. The assay was repeated three times. β‐Actin was taken as an internal control. The relative level of proteins was calculated by the ratio of the gray value of the target band to the internal reference band. Detailed information on relevant antibodies is presented in Table 2.

**TABLE 2:** Antibody information in western blot

Antibody	WB	Specificity	Company
β‐Actin	1:5000	Rabbit polyclonal	Abcam, China
NR5A2	1:1000	Rabbit polyclonal	Abcam, China
IgG	1:5000	Goat anti‐Rabbit	Abcam, China

### 2.8 Dual‐luciferase assay

To identify the targeting relationship between microRNA‐433‐3p and NR5A2, the 3′UTR of NR5A2 was cloned into a pcMV6 vector containing luciferase gene. The luciferase vector fused with wild‐type or mutant NR5A2 3′UTR was transfected into glioma cells together with NC mimic or microRNA‐433‐3p mimic with Lipofectamine 2000 (Invitrogen). In the end, luciferase activity was assayed with luciferase assay reagent (Promega, USA). Firefly luciferase was normalized to Renilla luciferase.

### 2.9 Cisplatin cytotoxicity test

Glioma cells were planted to 96‐well plate (1 × 10^4^ cells/well), and at 48 h after transfection, cisplatin with a final concentration of 0.5, 1.0, 2.0, 4.0, or 8.0 μg/ml was added to three replicate wells for 24 h. Cell viability was determined by MTT method. Next, 20 μl 5 mg/ml MTT was supplemented for 4 h of incubation at 37°C. A microplate reader was utilized to read the absorbance at 490 nm. All experiments were conducted at least three times.

### 2.10 Statistical analysis

All the data were processed by SPSS 22.0 statistical software. The analyzed results were presented as mean ± standard deviation, and the comparison of two groups was performed by *t*‐test, *p *< 0.05.

## 3 RESULTS

### 3.1 NR5A2 is increased in glioma cells and tissue

**FIGURE 1:**
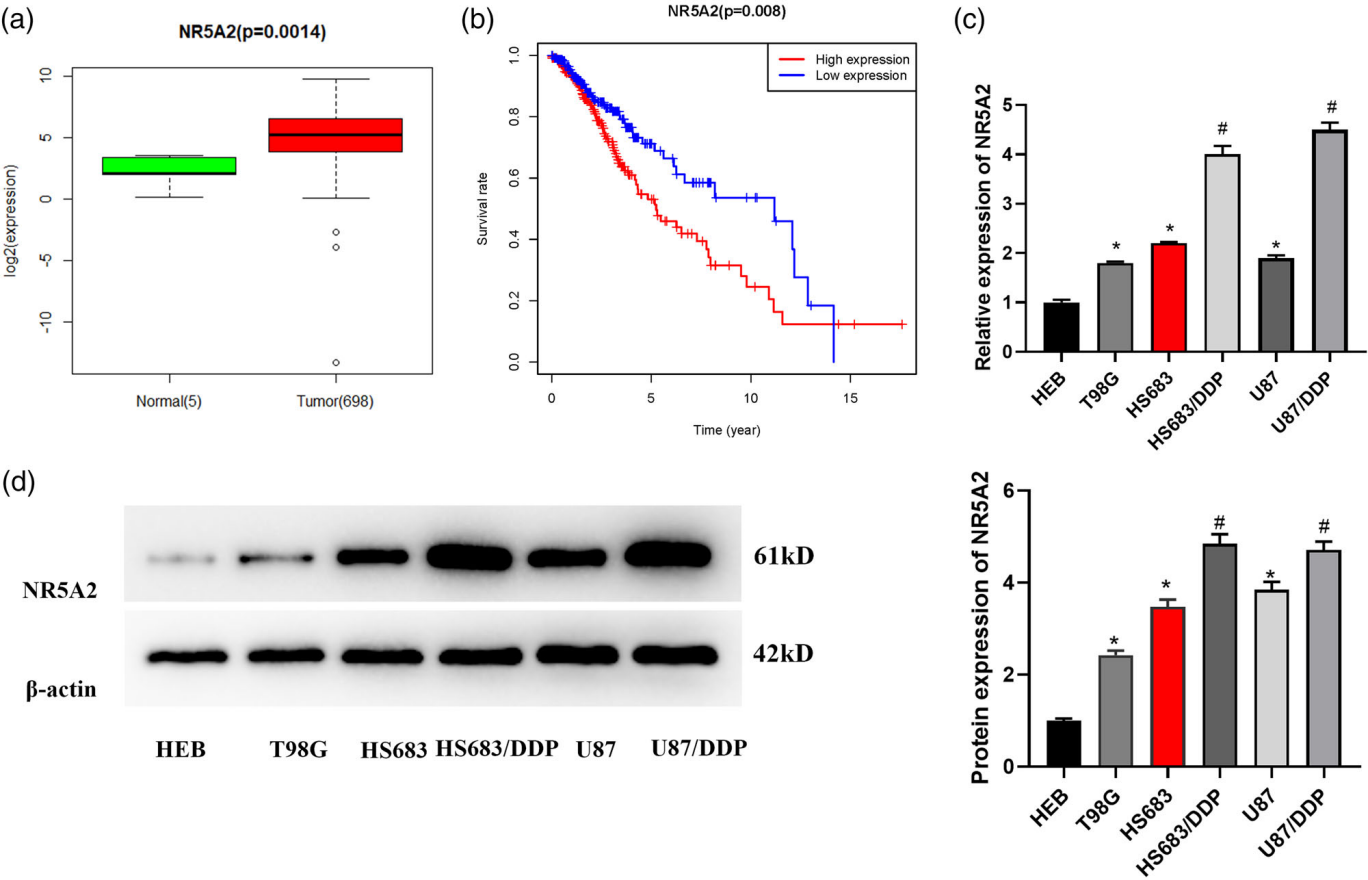
NR5A2 is increased in glioma cells and tissue. (a) Box plot of NR5A2 differential expression in TCGA; (b) the effect of NR5A2 on patient's survival detected by survival analysis; (c) NR5A2 mRNA level in HEB, HS683, T98G, and U87, cells and drug‐resistant glioma cell lines HS683/DDP and U87/DDP assayed through qRT‐PCR; (d) NR5A2 protein level in HEB, HS683, T98G, and U87 cells, and drug‐resistant glioma cell lines HS683/DDP and U87/DDP detected by western blot; * compared with HEB group, #compared with corresponding parent cells, *p *< 0.05

The glioma data downloaded from the TCGA database indicated that NR5A2 was significantly increased in glioma tissue (Figure 1a), and survival analysis result showed that patients with high NR5A2 expression in glioma suffered poor prognosis (Figure 1b). Several studies have revealed that NR5A2 plays a crucial role in cancer progression (Liu et al., 2019; Z. Luo et al., 2017). In this study, NR5A2 was taken as the object of interest, and its biological role in progression of glioma was clarified. qRT‐PCR and western blot were introduced to evaluate NR5A2 level in HEB, T98G, HS683, and U87 cell lines. NR5A2 was increased in glioma cells (*p *< 0.05, Figure 1c,d). In addition, we found that NR5A2 level in glioma resistant cell lines HS683/DDP and U87/DDP was conspicuously higher than that in its parental cells (*p *< 0.05, Figure 1c,d). Thus, NR5A2 was increased in glioma cells and tissue.

### 3.2 NR5A2 promotes the malignant progression of glioma

**FIGURE 2:**
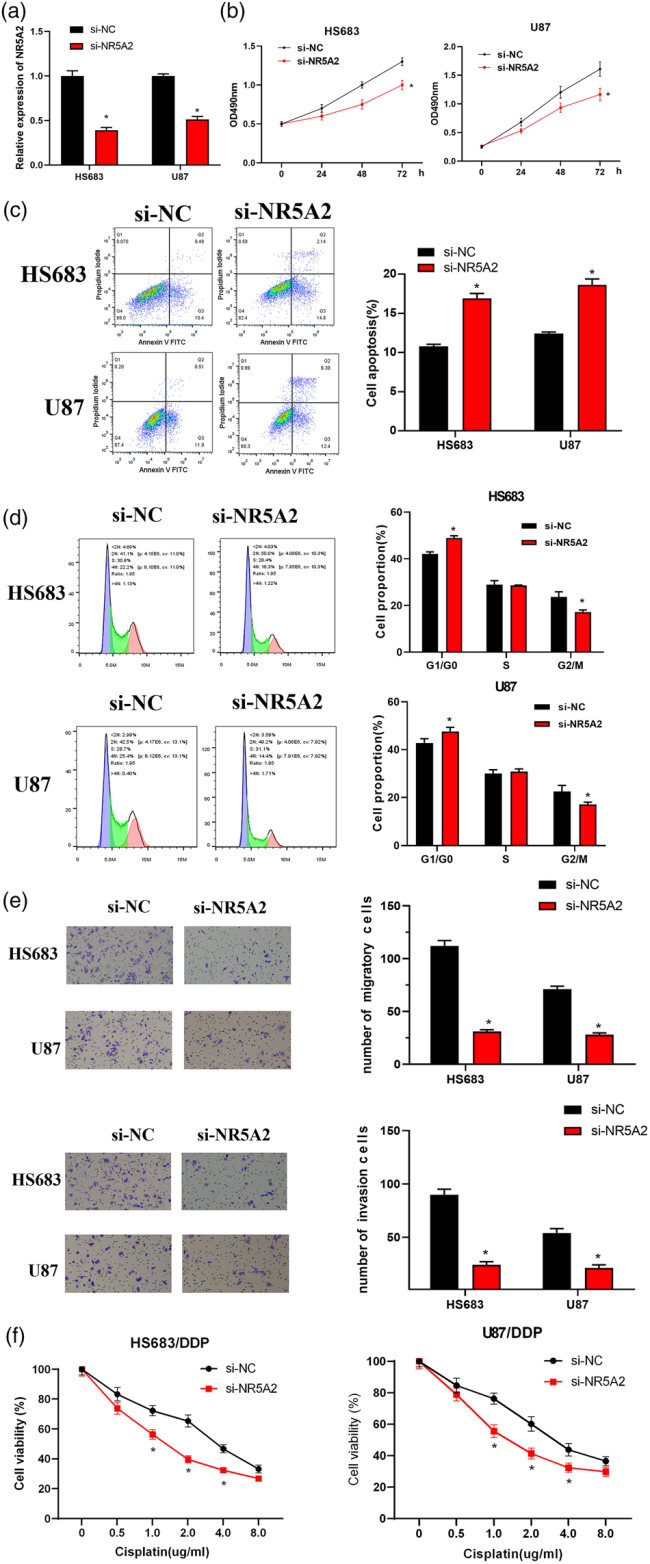
NR5A2 promotes the malignant progression of brain glioma. (a) Changes in NR5A2 expression in group si‐NC, si‐NR5A2 in HS683 and U87 cells, respectively, assayed through qRT‐PCR; (b) changes in the proliferative ability of glioma cells in different groups assessed by MTT assay; (c and d) changes in the apoptosis and cell cycle of glioma cells in various groups assessed by flow cytometry assay; (e) changes in the invasive and migratory abilities of glioma cells in various groups assessed by Transwell assay (100×); (f) response curve of HS683/DDP and U87/DDP cells to cisplatin dose assessed by MTT; *compared with si‐NC group, *p *< 0.05

si‐NR5A2 was transfected into glioma cells HS683 and U87, respectively. qRT‐PCR assay result disclosed that NR5A2 level was noticeably decreased after cells transfected si‐NR5A2 (*p *< 0.05, Figure 2a). MTT assay was used to assess the impact of NR5A2 on proliferative ability of glioma cells. The result showed that interference with NR5A2 expression prominently repressed HS683and U87 cell proliferation after transfection for 48 and 72 h (*p *< 0.05, Figure 2b). The effect of NR5A2 on cell apoptosis was investigated by flow cytometry assay, and the apoptosis rate was detected by flow cytometry at 48 h after transfection. Results showed that interference with NR5A2 significantly promoted apoptosis (*p *< 0.05, Figure 2c). Flow cytometry assay was also utilized to assess cell cycle at 48 h after transfection, and the result indicated that silencing NR5A2 induced HS683 and U87 cell arrest at G0/G1 phase, indicating that NR5A2 may accelerate cell cycle progress and promote cell proliferation (*p *< 0.05, Figure 2d). Hence, NR5A2 promoted growth of glioma cells. In addition, effects of NR5A2 on HS683 and U87 cell invasive and migratory abilities were assessed by Transwell assay. The result showed that interference with NR5A2 significantly inhibited cell invasion and migration (*p *< 0.05, Figure 2e). MTT assay was used to investigate the impact of NR5A2 on chemosensitivity of glioma cells to cisplatin. When the concentration of cisplatin was 1, 2, and 4 μg/ml, cell viability of HS683/DDP and U87/DDP with silencing NR5A2 was notably lower than that of the si‐NC group, showing that silencing NR5A2 noticeably increased chemosensitivity of glioma cells to cisplatin (*p *< 0.05). It could be seen that silencing NR5A2 hampered invasion and migration of glioma cells, and enhanced chemosensitivity to cisplatin. Thus, NR5A2 fostered malignant progression of brain glioma.

### 3.3 MicroRNA‐433‐3p targets and restrains the expression of NR5A2

**FIGURE 3:**
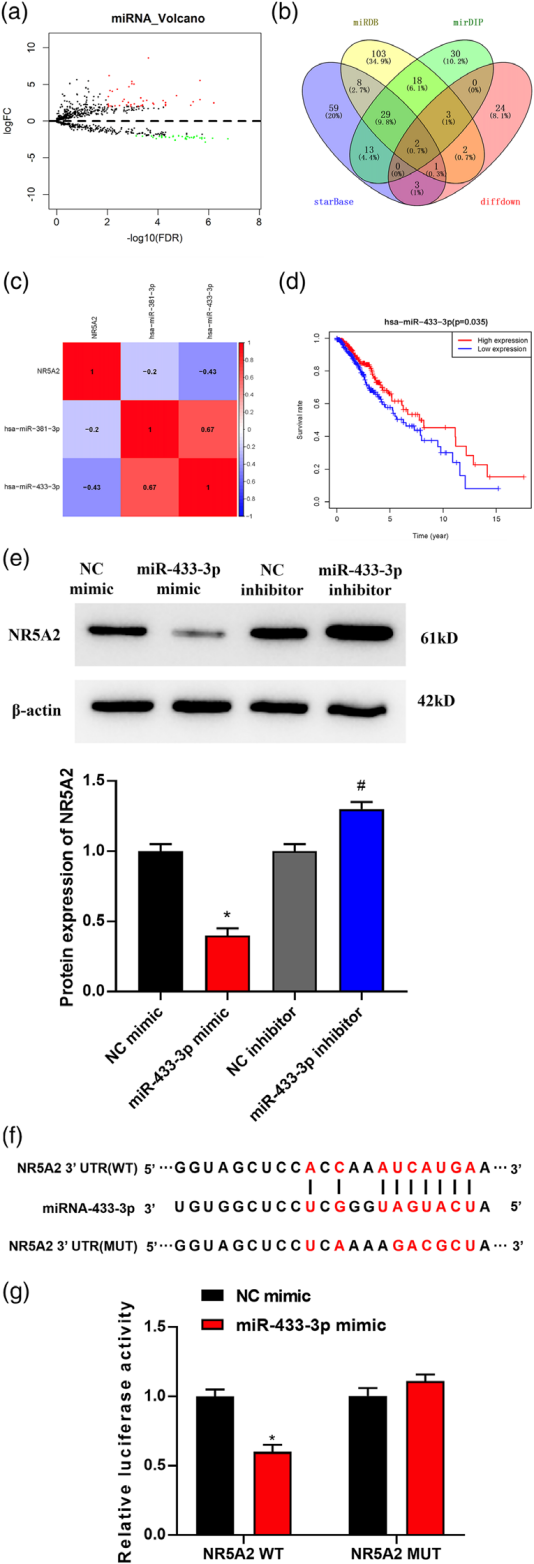
MicroRNA‐433‐3p targets NR5A2 to decrease NR5A2 level. (a) Volcano map of differential microRNAs in the glioma dataset of the TCGA database; (b) Venn diagram of the putative upstream microRNAs and the differential microRNAs; (c) Pearson correlation analysis of NR5A2 and its predicted upstream microRNAs; (d) survival curves show the effect of the expression of microRNA‐433‐3p on the prognosis of patients; (e) changes of NR5A2 protein and mRNA levels upon forced and silenced expression of microRNA‐433‐3p, respectively; (f) the binding sites of microRNA‐433‐3p on NR5A2 3′UTR were predicted; (g) dual‐luciferase activity was used to detect whether there is an interaction between microRNA‐433‐3p and NR5A2; * compared with NC mimic group, #compared with NC inhibitor group, *p *< 0.05

Bioinformatics method was used to find the microRNA with binding sites on NR5A2 mRNA. The result of the differential analysis performed by using of R package “edgeR” showed that a total of 78 differential microRNAs were obtained (43 upregulated and 35 downregulated) (Figure 3a). Upstream microRNA prediction for NR5A2 was performed using bioinformatics databases, and an intersection of the predicted upstream microRNAs with the 35 differentially down‐regulated microRNAs was taken to get two microRNAs with targeted binding sites to NR5A2 (microRNA‐433‐3p, microRNA‐381‐3p) (Figure 3b). Pearson correlation analysis revealed that NR5A2 and microRNA‐433‐3p had the highest Pearson correlation coefficient (Figure 3c), suggesting that microRNA‐433‐3p might be able to target NR5A2 to regulate the malignant progression of glioma. Meanwhile, Kaplan–Meier survival analysis showed that patients with high expression of microRNA‐433‐3p shared a better prognostic status (Figure 3d).

Western blot and qRT‐PCR results suggested that forced microRNA‐433‐3p level hindered NR5A2 level, while interference with microRNA‐433‐3p promoted NR5A2 expression, with statistical significance (*p *< 0.05, Figure 3e). Luciferase reporter vectors containing NR5A2 3′UTR fragments were transfected into HS683 cells with microRNA‐433‐3p mimic or NC mimic. Different 3′UTR fragments were used to validate their putative functional sites, including wild‐type and mutant 3′UTR (Figure 3f). Dual‐luciferase assay result indicated that forced microRNA‐433‐3p level hindered luciferase activity of the cells with wild‐type NR5A2 3′UTR but had no impact on that of cells with mutant NR5A2 3′UTR, with statistical significance (*p *< 0.05, Figure 3g). Hence, microRNA‐433‐3p directly targeted NR5A2 3′UTR to reduce NR5A2 expression.

### 3.4 MicroRNA‐433‐3p represses malignant progression of glioma through binding NR5A2

**FIGURE 4:**
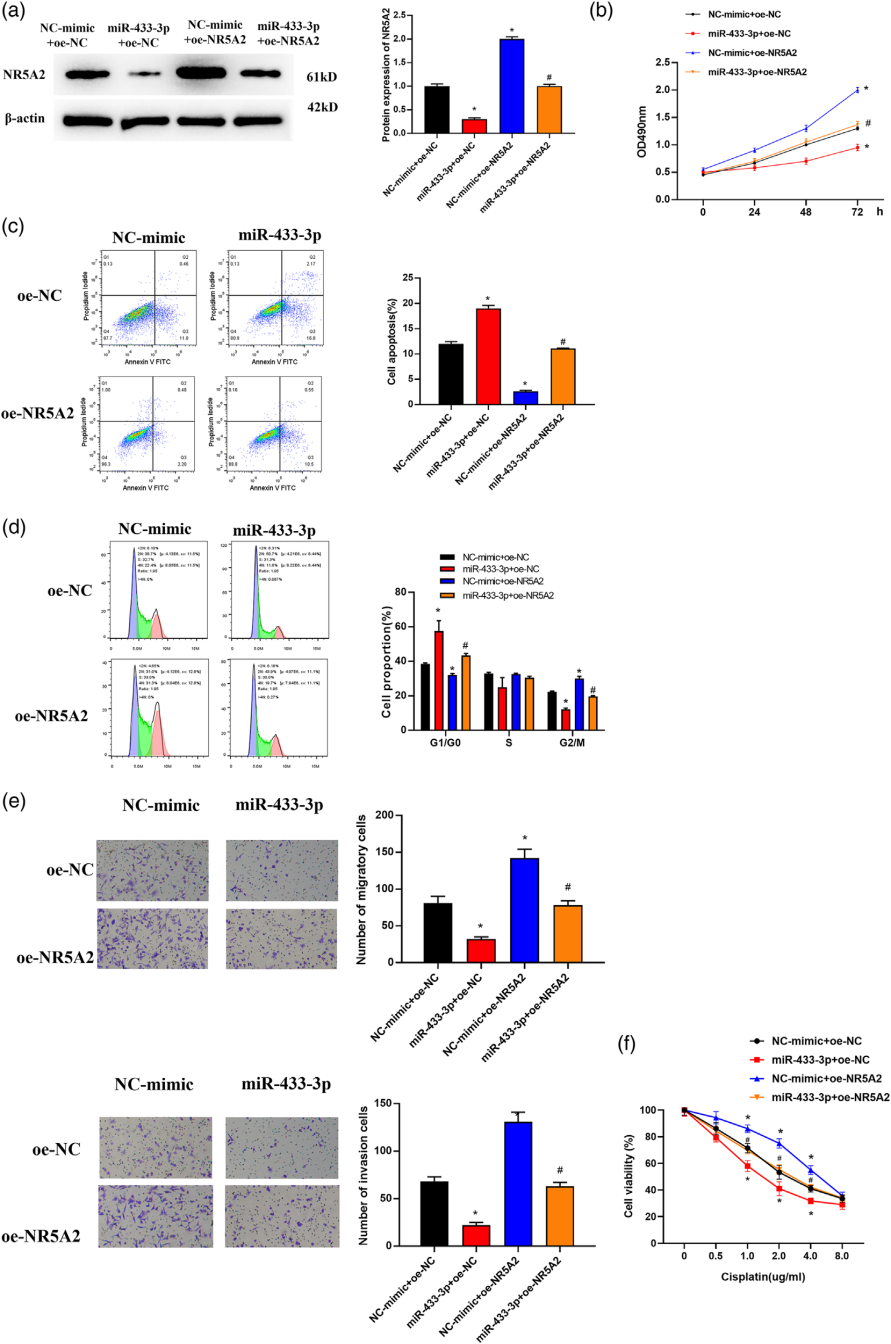
MicroRNA‐433‐3p hampers malignant progression of glioma via binding to NR5A2. (a) Changes of NR5A2 protein and mRNA levels in transfection groups detected by western blot and qRT‐PCR; (b) the impact of simultaneous overexpression of microRNA‐433‐3p and NR5A2 on proliferation of glioma cells detected by MTT assay; (c and d) the effect of simultaneous forced microRNA‐433‐3p and NR5A2 on apoptosis level and cell cycle of glioma cells assayed by flow cytometry; (e) the impact of simultaneous overexpression of microRNA‐433‐3p and NR5A2 on invasion and migration of glioma cells assayed by Transwell assay (100×); (f) response curve of U87/DDP cells to cisplatin dose assessed by MTT; *microRNA‐433‐3p+oe‐NC group and NC‐mimic+oe‐NR5A2 group compared with NC‐mimic+oe‐NC group, respectively, #NC‐mimic+oe‐NR5A2 group and microRNA‐433‐3p+ oe‐NR5A2 group compared with microRNA‐433‐3p+oe‐NC group, *p *< 0.05

To investigate the effect of microRNA‐433‐3p targeting NR5A2 on glioma cells, NR5A2 overexpression vectors were constructed, and the vectors (or empty vectors) were cotransfected with microRNA‐433‐3p mimic (or NC mimic) into HS683 cells. Western blot and qRT‐PCR assays confirmed that forced NR5A2 level rescued the repressive impact of microRNA‐433‐3p on NR5A2 (*p *< 0.05, Figure 4a). MTT assay displayed that forced microRNA‐433‐3p level conspicuously repressed increased cell proliferation induced by NR5A2 (*p *< 0.05, Figure 4b). Flow cytometry result exhibited that overexpression of microRNA‐433‐3p notably reverses inhibition of NR5A2 on cell apoptosis (*p *< 0.05, Figure 4c), increases tumor cells in G0/G1 phase, delays cell cycle progression, and inhibits cell proliferation (*p *< 0.05, Figure 4d). Transwell assay result denoted that overexpression of microRNA‐433‐3p noticeably hampered cell invasion and migration induced by NR5A2, and to some extent rescued promoting impact of NR5A2 on the cell invasion and migration of glioma (*p *< 0.05, Figure 4e). Cisplatin chemosensitivity assay showed that forced expression of microRNA‐433‐3p in HS683/DDP cell line markedly promoted sensitivity of glioma cells to cisplatin, while NR5A2 could reverse the promotion of microRNA‐433‐3p on glioma cell sensitivity to cisplatin (Figure 4f). The above experimental results indicated that microRNA‐433‐3p repressed NR5A2 level and thus inhibited malignant progression of glioma.

## 4 DISCUSSION

NR5A2 belongs to the superfamily of nuclear receptors (Z. Luo et al., 2017) and is crucial in many biological processes. It has been found that NR5A2 can accelerate the development of intestinal tumors through regulating cell cycle and inflammation (Petruzzelli et al., 2016) and promote the invasive ability of breast cancer cells via changing actin cytoskeleton or reducing the transcription of cyclin‐dependent kinase inhibitors independent of ERα and p53 state (Bianco et al., 2015; Chand et al., 2010). In addition, NR5A2 is a direct target for microRNA‐376c to inhibit cell proliferation and invasion via Wnt signaling pathway in nonsmall cell lung cancer (Jiang et al., 2016). Liang et al. (2015) illustrated that microRNA‐381 can promote progression of colon cells by upregulating NR5A2. Our study showed that NR5A2 was increased in glioma cells. Moreover, silencing NR5A2 hindered cell cycle progress, proliferation, invasion, and migration of glioma cells and induced cell apoptosis. In addition, NR5A2 can modulate chemosensitivity of cancer cells to cisplatin. For example, forced expression of NR5A2 weakens the cytotoxicity of cisplatin in breast cancer cells and xenograft models (S. Wang et al., 2018). We manifested that NR5A2 was activated in drug‐resistant glioma cell line while silencing NR5A2 notably inhibiting cell resistance to cisplatin. As such Q. Yang, Deng, et al. (2020) manifested that aberrant level of NR5A2 results in unfavorable clinical prognosis and overall survival of patients, and upregulation of NR5A2 enhances cell resistance to temozolomide glioma. Collectively, NR5A2 could facilitate glioma cell malignant progression and enhance cell resistance to cisplatin.

Many microRNAs have been proved to have positive or negative effects on brain glioma (Geng et al., 2019). For example, microRNA‐499a‐5p fosters development of glioma via regulating the expression of KPNA2 (Z. Yang, Li, et al., 2020). MicroRNA‐30a inhibits self‐renewal of glioma stem cells through blocking NT5E‐dependent Akt signaling (Peng et al., 2020). MicroRNA‐433 plays different roles in the progression of human tumors. MicroRNA‐433 is decreased in gastric cancer (H. Luo et al., 2009), lung cancer (Li et al., 2019), visceral adipose tissue of patients with nonalcoholic fatty liver disease (Estep et al., 2010), and hepatocellular carcinoma with hepatitis B virus infection (W. Wang et al., 2012). In addition, microRNA‐433 negatively modulates 5‐fluorouracil‐sensitive thymidylate synthetase level in HeLa cells (Gotanda et al., 2013). Moreover, microRNA‐433‐3p is implicated with cancer cell drug resistance. For example, binding of NORAD and microRNA‐433‐3p induces gastric cancer cell resistance to oxaliplatin (J. Wang et al., 2021). Nonetheless, the exact role of microRNA‐433 in glioma is less studied. Herein, we proved that the expression of NR5A2 was negatively correlated with that of microRNA‐433‐3p in glioma and confirmed that NR5A2 was the direct target of microRNA‐433‐3p by luciferase reporter assay. We further investigated that microRNA‐433 was decreased in glioma cells. After upregulating microRNA‐433‐3p level in glioma cell line U87, microRNA‐433‐3p decreased NR5A2 level. MicroRNA‐433‐3p plays a tumor repressor role in glioma by targeting NR5A2, thus regulating cell behaviors, and enhancing chemosensitivity to cisplatin. As such, a study described that microRNA‐433‐3p is underexpressed in glioma tissue and cells and activates apoptotic‐signaling pathway to enhance glioma chemosensitivity to temozolomide by targeting CREB (Sun et al., 2017). Thus, microRNA‐433‐3p may enhance chemosensitivity of glioma to cisplatin via modulating cell cycle distribution, activating cell apoptosis, and downregulating NR5A2 level.

Overall, our study unveiled that microRNA‐433‐3p may be a possible repressive gene in glioma. MicroRNA‐433‐3p hindered malignant progression of glioma via binding NR5A2 and enhanced glioma chemosensitivity to cisplatin. This discovery may lay groundwork for management of brain glioma. Despite the finding that microRNA‐433‐3p could restrain glioma progression via targeting NR5A2, other targets of microRNA‐433‐3p may also affect the carcinogenesis of gliomas. The molecular mechanism and signaling pathway of microRNA‐433‐3p in glioma, therefore, need to be further studied.

## CONFLICT OF INTEREST

The authors declare no conflict of interest.

## AUTHOR CONTRIBUTIONS

Jun Li contributed to the study design. Jingshun Gu and Juntong Wang conducted the literature search. Aiwu You and Yuyan Zhang acquired the data. Guoming Rao and Shuang Li wrote the article. Xuehua Ge and Kun Zhang performed data analysis. Jun Li revised the article. Dongchun Wang gave the final approval of the version to be submitted.

## Data Availability

The data and materials in the current study are available from the corresponding author upon reasonable request.
